# Pediatric claims in Italy during a 8-years survey

**DOI:** 10.1186/1824-7288-40-S1-A87

**Published:** 2014-08-11

**Authors:** Rino Agostiniani, Antonio Correra, Paolo D’Agostino, Ernesto D’Aloja, Luigi Greco, Paolo Tagliabue, Vassilios Fanos

**Affiliations:** 1 Neonatal Intensive Care Unit, Puericulture Institute and Neonatal Section, AOU and University of Cagliari, Italy

## 

Very few data are available on pediatrics malpractice claims. We report the first data obtained in Italy on pediatrics regarding a wide population study during a 8 years survey. Data concerning 164 claims are presented and discussed. Our data suggest how big is the problem and they may be helpful to face it.

## Introduction

Pediatrics is not a high-risk specialty in terms of the number of claims, although some of the largest financial payouts have been for multiple disabled children with perinatal injuries and long life expectancy [1-5]. We report the first data obtained in Italy on pediatrics regarding a wide population study.

## Materials and methods

We conducted a retrospective, descriptive analysis of a nation-wide database on pediatric malpractice claims, in which patients alleged a permanent impairment related to a medical misconduct. The Italian Society of Pediatrics (Società Italiana di Pediatria; SIP) has developed a link – thorough insurance broker Willis Italian SpA – with an insurance company (CARIGE Assicurazioni SpA) that insures a wide proportion of Italian pediatricians (nearly 60% out of 8000 physicians).

We asked Willis to perform a query of its database, looking at malpractice claims reported between January, 1^st^ 2005 and December, 31^st^ 2012 involving pediatrics while avoiding neonatology.

Definitions are used as previously reported by ours [6].

## Results

We found 164 claims, the majority of which were reported in the last two years (year 2011: n=65; year 2012: n=35), covering more than 2/3 of the total number of claims. 89 were from South Italy, 43 form the north and 32 from Central Italy. 141 involved the public health system, 13 the private health system and 8 family pediatricians. 102 were criminal actions, 53 civil actions, 5 mixed actions and 4 cautelative claims. We found 89 death claims and 65 claims for permanent impairment. Each claim interested one or more physicians. Main areas of class are presented in table 1. Among surgical pathologies, 6 were gastrointestinal and 6 involved testis. Among infections, 6 were pleura-pulmonary diseases, 5 fulminant sepsis (2 meningococcal), 3 tuberculosis (2 meningeal, 1 pulmonary), 1 *Salmonellosis*, 1 *Candidosis*, 1 chicken pox, 1 scarlet fever.

**Table 1:** Main area of claims in the 8 years-survey

Main area of claims	Number of claims
Surgical pathology	26

Infections	20

Gastrointestinal medical problem (vomiting, diarrhea) with subsequent dehydration	14

Iatrogenic pathology (drugs, catheters…)	5

Undiagnosed tumor	4

Undiagnosed congenital cardiopathy	3

Post vaccinal encephalitis	3

Undiagnosed urinary disease	2

Undiagnosed rare disease	2

Undiagnosed deafness	2

## Conclusions

Malpractice data can be used to identify problem-prone clinical processes and suggest interventions that may reduce errors. Continual medical education should be oriented in areas of claims also improving physician communication skills [7].
